# A new computational methodology for the characterization of complex molecular environments using IR spectroscopy: bridging the gap between experiments and computations†

**DOI:** 10.1039/d4sc03219e

**Published:** 2024-08-13

**Authors:** Laura X. Sepulveda-Montaño, Johan F. Galindo, Daniel G. Kuroda

**Affiliations:** a Department of Chemistry, Louisiana State University Baton Rouge Louisiana 70803 USA dkuroda@lsu.edu; b Department of Chemistry, Universidad Nacional de Colombia Sede Bogotá Bogotá 111321 Colombia

## Abstract

The molecular interactions and dynamics of complex liquid solutions are now routinely measured using IR and 2DIR spectroscopy. In particular, the use of the latter allows the determination of the frequency fluctuation correlation function (FFCF), while the former provides us with the average frequency. In turn, the FFCF can be used to quantify the vibrational dynamics of a molecule in a solution, and the center frequency provides details about the chemical environment, solvatochromism, of the vibrational mode. In simple solutions, the IR methodology can be used to unambiguously assign the interactions and dynamics observed by a molecule in solution. However, in complex environments with molecular heterogeneities, this assignment is not simple. Therefore, a method that allows for such an assignment is essential. Here, a parametrization free method, called Instantaneous Frequencies of Molecules or IFM, is presented. The IFM method, when coupled to classical molecular simulations, can predict the FFCF of a molecule in solutions. Here, *N*-methylacetamide (NMA) in seven different chemical environments, both simple and complex, is used to test this new method. The results show good agreement with experiments for the NMA solvatochromism and FFCF dynamics, including characteristic times and amplitudes of fluctuations. In addition, the new method shows equivalent or improved results when compared to conventional frequency maps. Overall, the use of the new method in conjunction with molecular dynamics simulations allows unlocking the full potential of IR spectroscopy to generate molecular maps from vibrational observables, capable of describing the interaction landscape of complex molecular systems.

## Abstract 2

The new instantaneous frequency method presented here can be integrated with molecular dynamics simulations to link the classical atomistic representation of the system to its vibrational observables.

## Graphical abstract

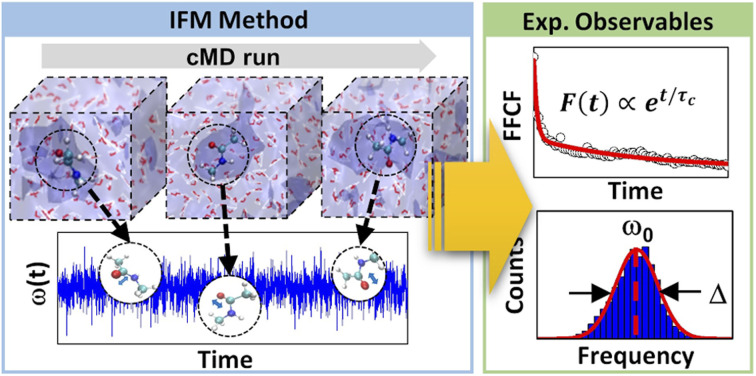

## Introduction

**Fig. 1:**
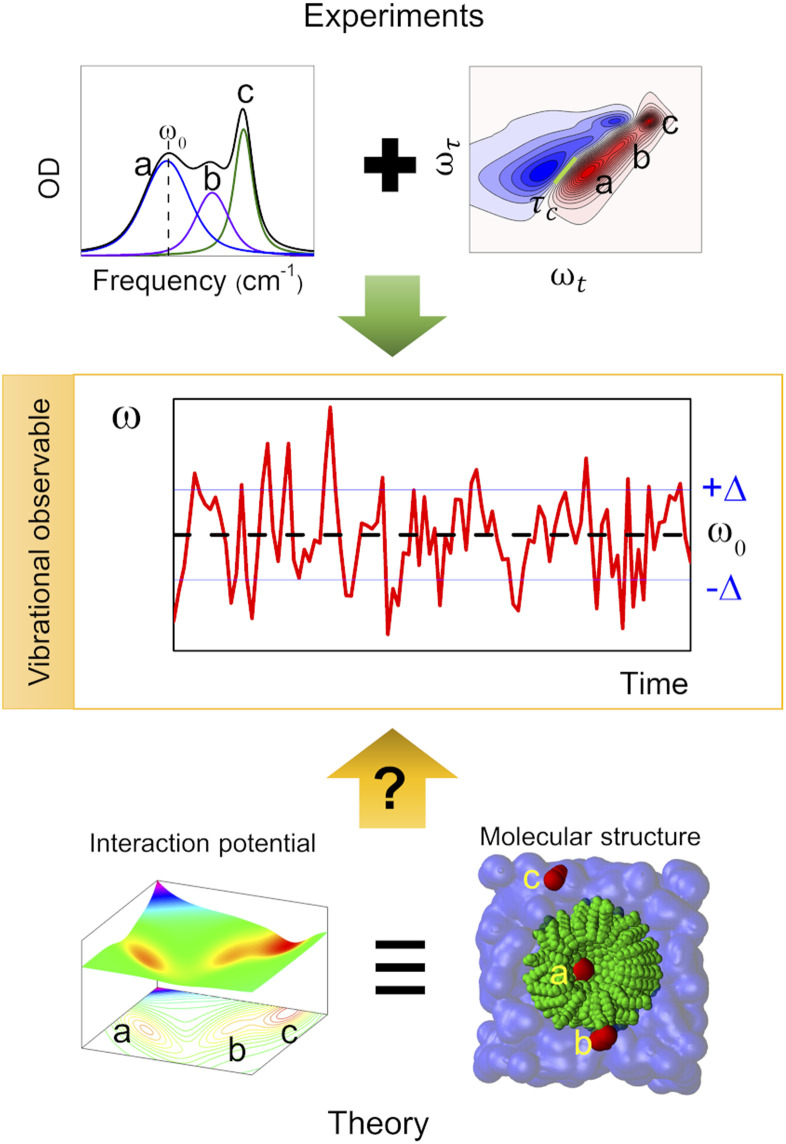
Use of vibrational observables to study the PES and chemical environment of a molecular system.

Complex liquid solutions are multi-component systems with intricate molecular arrangements arising from the diverse interactions among the components in the mixture. Due to the large number of contributions to the potential energy surface (PES), complex molecular systems typically exhibit nanoscopic heterogeneities, especially in cases where the solution contains molecules with contrasting chemical moieties, such as polar and non-polar.^1^ Therefore, the complex and non-ideal behaviour of the liquid creates challenges associated with its characterization, both experimentally and computationally. Moreover, the liquid state of the mixture allows its molecular components to diffuse freely (translate and rotate), resulting in a chemical environment that varies as a function of time. Consequently, one can characterize the structure and dynamics of the complex solution to indirectly assess the PES since these two properties are governed by its energy landscape (Fig. 1).

The development of methods that can generate a map of intermolecular forces based on the dynamics and interactions of each component in the mixture has been a long-standing goal for understanding and later tailoring complex molecular systems. Experimentally, the complete characterization of complex solutions requires the use of techniques capable of resolving the individual interactions occurring in the multicomponent system. Among the various experimental methods commonly used to characterize the molecular structure of solutions, one can find NMR, UV-vis, fluorescence, Raman and IR spectroscopies as well as scattering techniques. For example, NMR has been successfully applied to study the diffusion of species in complex solutions, the molecular structure of DNA and RNA in live human cells,^2^ and the isomerization dynamics in ionic liquids.^3^ However, NMR spectroscopy lacks the required time resolution to characterize solutions undergoing ultrafast dynamics with characteristic times faster than nanoseconds, such as liquids at room temperature, and yields averaged signals for these processes.^4^ UV-vis and fluorescence spectroscopies have also shown potential for the analysis of micellar systems,^5^ for probing the chemical environment of hydrocarbons,^6^ and for exploring the air/liquid interphases.^7^ Notwithstanding the capabilities of these two spectroscopies, they are limited by the number of substrates with responses in the UV-vis spectral range, which significantly reduces the number of complex solutions that can be analysed with them.

Scattering techniques, such as X-ray and neutron scattering, have also been used to characterize complex solutions such as deep eutectic solvents,^1,8–10^ ionic liquids,^11–13^ and solutions of proteins and DNA.^14–16^ These techniques provide structural information at the atomic level through the scattering profile. For a liquid sample, the peaks of the scattering profile contain different atomic contributions, requiring a nontrivial deconvolution to elucidate the structure from the data.^17^ To this end, the scattering experiments usually rely on molecular dynamics (MD) simulations to model and evaluate the different atomic contributions to each peak.^9,12,13^ Hence, the correct modelling of scattering data requires a good parameterization of the MD force field since two different atomistic models can generate the same scattering profile.^18^

An alternative characterization method for complex solutions relies on the use of vibrational spectroscopy, which includes both IR and Raman spectroscopies. By analysing the frequency shift of the vibrational bands in the IR or Raman spectrum, it is possible to probe the chemical environment.^19–22^ In either case, the vibrational bands are related to the molecular vibrational modes, either through the dipole (IR)^23^ or the polarizability (Raman)^24^ of the molecule; consequently, the bands change as a function of the molecular environment.

Normal modes are easily calculated from the motions in a potential energy surface, but the dipole moment associated with a vibrational transition is simpler than the equivalent polarizability tensor, which requires full electronic structure calculations.^25,26^ Hence, the changes in the Raman bands are often difficult to relate to specific molecular environments.

Normal modes are mathematical entities related to the diagonalization of the Hessian matrix (*i.e.*, second derivatives of the PES at the minimum) and represent the motion of collections of atoms in a molecule. More importantly, the IR band can be understood in terms of normal modes using the dipole moment formalism.^23^ Similar to the PES, the normal mode frequencies (PES curvature) are highly dependent on the molecular speciation and environment (*i.e.*, structure, temperature, pressure, molecular interactions, *etc.*).^19,27,28^ Therefore, the molecular environment of a molecule can be, and is, typically deduced from the central frequency of an IR band associated with a particular vibrational mode. In addition, the time evolution (dynamics) of the vibrational modes in a molecular system can be characterized using time resolved vibrational spectroscopy. Among the various possible time resolved methods, 2DIR spectroscopy is the most powerful for describing the dynamics of the system at thermal equilibrium using specific sets of atoms, such as carbonyl groups.^29^ Among other features, 2DIR spectroscopy measures the dynamics of the frequency-fluctuation correlation function (FFCF), or equivalently, the time scale at which the system loses its memory of a given molecular environment.^27^ In particular, the FFCF provides information about the interaction potential among the molecules through their dynamics.^30^ Moreover, the FFCF contains information about the various chemical environments through its amplitude term because it is the variance of the distribution of the frequencies (environments) sensed by the molecules in the sample.^27^

The use of 2DIR spectroscopy to characterize the molecular environments in a complex solution is also challenging due to the lack of prior knowledge of the chemical environment and interactions affecting the molecule under study. Thus, to obtain the location and interaction landscape of the molecular components *via* 2DIR spectroscopy, the modelling of the system using MD simulations is often required. However, the MD simulation does not directly provide the vibrational observables, and instead these are computed using the so-called frequency maps.

Frequency maps transform molecular coordinates into specific variables, such as electric fields, to obtain the instantaneous frequency of a molecule in the MD simulation.^31^ Hence, frequency maps have been instrumental in understanding and modelling vibrational spectroscopy data at a low computational cost. However, the applicability of this tool is generally limited to specific molecular systems for which the map was originally parametrized, and its extension to “new” chemical environments often requires further parametrization.^32–35^ Moreover, the intricacies in the theoretical description of complex molecular environments with nanoscopic heterogeneities do not always guarantee the access to such modelling and its transformation into vibrational observables, such as frequency shifts. In order to facilitate the experimental analysis of the IR spectroscopic data (IR position, line shape and dynamics), a new computational methodology is needed. In particular, this methodology should not only accurately translate molecular arrangements (environments) of highly heterogeneous molecular systems into frequency fluctuations but also be computationally efficient.

**Fig. 2:**
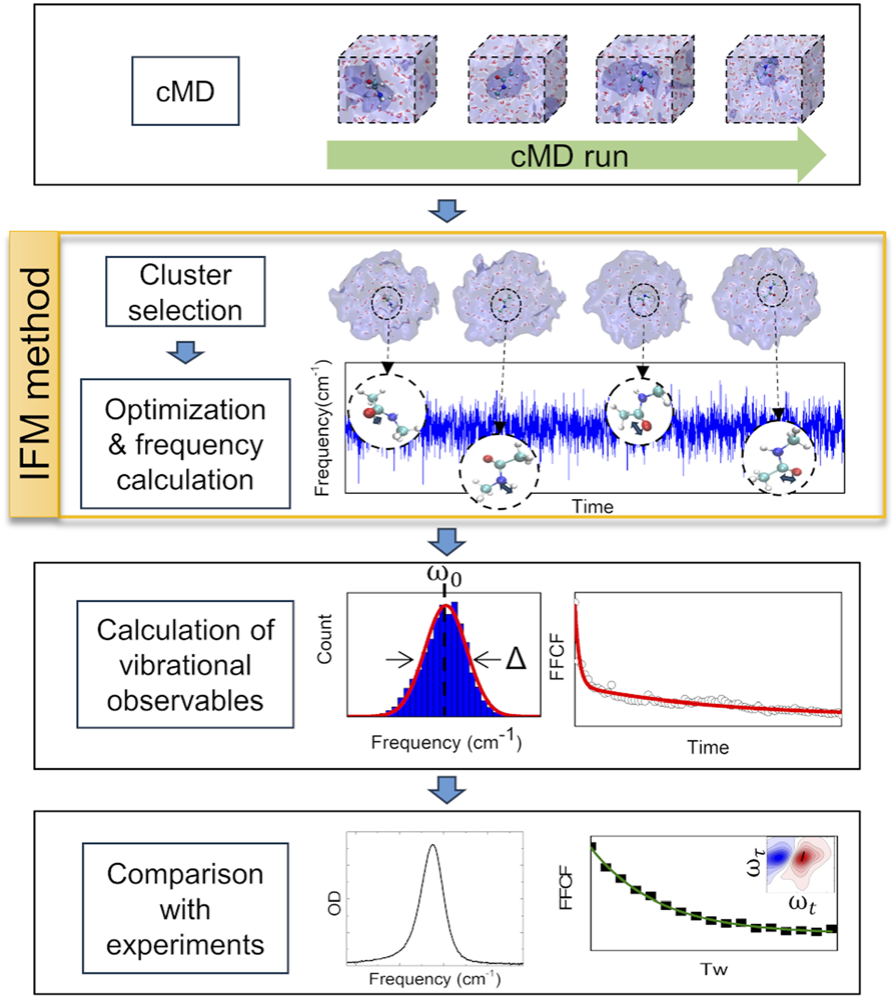
Procedure followed for the development and testing of the IFM method.

In this work, a new methodology (Instantaneous Frequencies of Molecules, IFM) is presented. This method uses a quantum mechanical method to compute the instantaneous frequencies of the molecules in a given molecular environment (Fig. 2). The exponential growth of computational power has allowed the direct use of *ab initio* quantum chemical and semiempirical methods to calculate frequencies. However, the proper description of the vibrational frequencies of a solvated molecule usually requires multiple solvation shells beyond the first one.^31^ Hence, a low cost computational method is needed to calculate the vibrational frequencies of such large systems, typically containing more than 100 atoms.

GFN2-xTB is a new tight binding approach that uses multipole expansion and density-dependent dispersion fluctuation to describe electrostatic interactions.^36,37^ In particular, this semiempirical method is tailored to accurately compute the geometries, non-covalent interactions, and vibrational frequencies of molecular systems and has low computational cost.^36,37^ Furthermore, it has recently been shown that GFN2-xTB correctly predicts the vibrational solvatochromism of various molecular probes typically used in IR spectroscopy, depending only on the correct description of the solute and its chemical environment as defined by the classical force field.^20^ This makes IFM calculations fully transferable to complex molecular arrangements without the need of new parameterization, beyond the selection of an appropriate classical representation (*i.e.*, force field).

The applicability of the IFM method for vibrational spectroscopy is tested on a molecular system composed of *N*-methylacetamide (NMA) in different solvents, ranging from simple to complex. NMA was selected because of its amide I vibrational mode, which is highly sensitive to the chemical environment, as evidenced by its solvatochromic frequency shifts in different solvents.^38–40^ In addition, as a model peptide unit, NMA has been extensively studied both experimentally and computationally. From a theoretical perspective, there are now many MD simulation studies dedicated to the solvation structure and dynamics of this molecule and some provide frequency maps to correlate with vibrational dynamics studies.^32,41–44^ From an experimental perspective, the vibrational dynamics of NMA has been thoroughly investigated using time resolved vibrational spectroscopy,^32,33,41,45–47^ making it an excellent reference for the IFM method. To this end, the following systems have been selected: NMA in heavy water (D_2_O), chloroform (CLF), dimethylsulfoxide (DMSO), tetrahydrofuran (THF), and toluene (TOL), as well as mixtures of DMSO and THF and of DMSO and D_2_O. The suitability of the computational methodology combining MD simulations and the IFM method (MD-IFM) was evaluated by computing and comparing with experimental data the vibrational frequency shifts (solvatochromism), the FFCF dynamics (solvent motions), and the amplitudes of the frequency fluctuations (distribution of environments) of NMA in different molecular environments.

## Methods

The experimental measurements to test the IFM method in the present work include linear and non-linear IR spectroscopy. The procedures and specifications can be found in the ESI.†

The schematic computational methodology for the IFM calculation is described in Fig. 2. However, a brief description is given in the following sections, and an example of the code used here can be found free of charge at https://github.com/dkurodalab/IFM.

### Molecular dynamics

MD simulations were carried out for the systems of NMA in D_2_O, CLF, DMSO, THF, TOL, DMSO : THF (1 : 3), and DMSO : D_2_O (1 : 1). In the first and last cases (D_2_O and D_2_O : DMSO (1 : 1) as solvents), the hydrogen atom attached to the nitrogen atom of NMA was replaced by a deuterium. Solvent boxes of 40 Å with or without the solute were built using PACKMOL.^48^ The quality of the force field was evaluated from the densities determined from an MD simulation in the pure system. MD simulations were performed using the AMBER 18 computational package.^49^ For water and all the other solvents, the TIP3P model and Generalized Amber Force Field (GAFF) were used, respectively.^50^ The NMA molecule was parameterized with the ff14SB force field.^51^ The MD simulation was performed by starting with an energy minimization, followed by a heating step from 0 to 300 K over 20 ps; the production run was carried out for 250 ps in the NPT ensemble after an equilibration run of 20 ns. A time step of 0.5 fs was used for all simulation stages. For the NMA/TOL system, a 1 ns production run was performed to accomplish the necessary averaging in the FFCF calculation.

### Frequency calculations and FFCF

Snapshots were selected every 50 fs from the MD production run and subsequently trimmed to the number of solvation shells needed for optimization and frequency calculations. At least two solvation shells were selected in each case, as they are sufficient to yield reliable frequency values (see the ESI†). To preserve the distribution of states from the MD, the geometry energy minimizations were performed only for the NMA molecule embedded in the selected solvation shells using the GFN2-xTB package. The geometry optimization and frequency calculations were carried out at the GFN2-xTB level of theory. The central frequencies for the amide I normal mode of NMA in different solvents represent the intensity weighted average. Note that the selection of GFN2-xTB semiempirical method is based on the low computational cost when compared to other *ab initio* methods, such as DFT (see the ESI†).

The FFCF was calculated from the instantaneous amide I frequencies over time: *C*(*t*) = *δω*(*t*)*δω*(0), where δ*ω*(*t*) is the delta between the frequency at time *t* and the average frequency (*ω*(*t*)–〈*ω*〉).^27^ The correlation time (*τ*_c_) was obtained by modelling the calculated FFCF with the Kubo formalism (*i.e.*, the bi-exponential decay function, see the ESI† for the fits of all molecular systems). The amplitude of the fluctuations was obtained by analysing the frequency distributions with Gaussian profiles.^27^

## Results and discussion

**Fig. 3:**
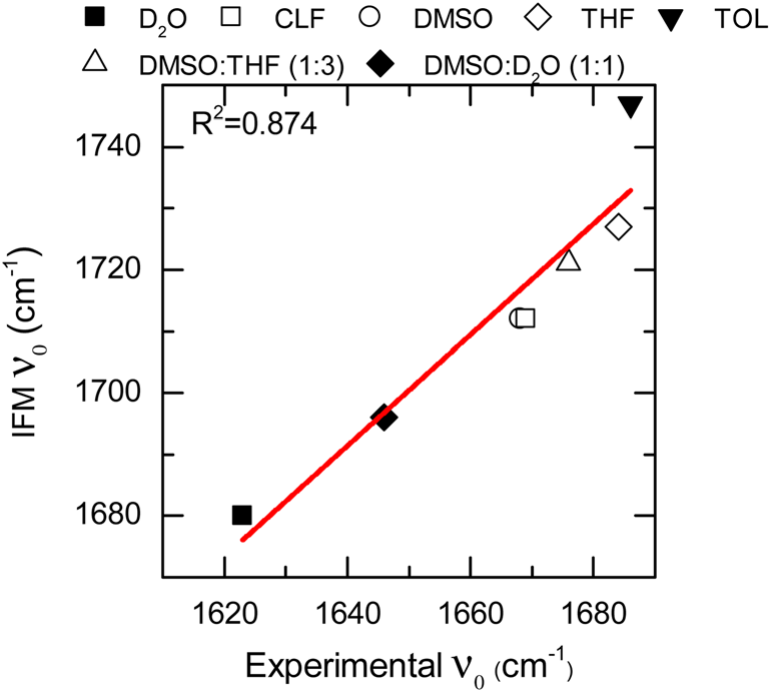
Solvatochromic shift for the NMA amide I mode in different solvents calculated through the IFM method *vs.* experimentally through FTIR (data points). Linear fit correspond to the red line.

The IFM method predicts Gaussian distributions for the amide I frequencies of NMA in different solvents, consistent with the stochastic motions of the molecular environment (see the ESI†).^52^ Because of the nature of the amide I vibrational mode, these distributions contain information about the molecular environment through their central frequencies; *i.e.*, solvatochromism.^28,43,53^ The central frequencies predicted by the IFM method correctly reproduce the experimental solvatochromic shift of NMA amide I mode in different solvents including mixtures. Moreover, a linear relationship is observed between the computed and experimental values (Fig. 3). A linear model of frequencies also shows that the IFM method overestimates their values by ∼60 cm^−1^. This deviation from experimental values is associated with the semiempirical method.^20^ A Pearson correlation coefficient (*R*^2^) of 0.874 is observed, indicating not only the strong linear correlation between the predicted and experimental frequency values but also the suitability of the IFM model for computing instantaneous frequencies. Notably, the NMA/TOL system deviates significantly from the linear trend. The linear model without NMA–toluene has a significantly better predictive power (*i.e.*, *R*^2^ increases to 0.990, see the ESI†). Note that the deviation in the prediction of the vibrational solvatochromism of amide I of NMA in toluene is likely due to a deficiency in the force field describing toluene in the MD simulation and not in the method. This assertion based on the GFN2-xTB method correctly characterizes other complex solvents, such as DMSO : D_2_O, where various and competing interactions are found.^28,45,54–58^

**Fig. 4:**
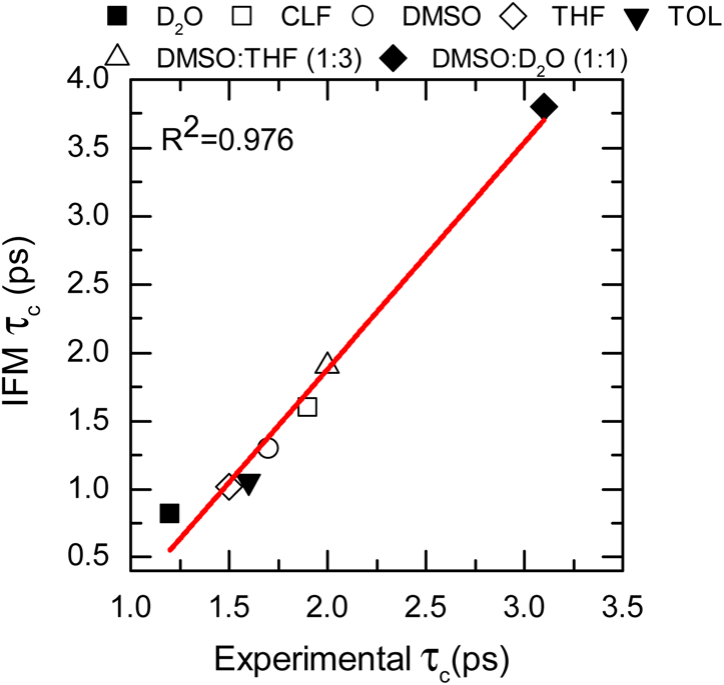
Correlation times calculated from the IFM method *vs.* the experimental values determined from 2DIR (data points). Linear fit corresponds to the red line.

To further assess the suitability of the computational methodology (MD-IFM), the FFCF was computed from the instantaneous frequencies predicted by the IFM method. Assuming a Kubo stochastic model,^27^ the FFCF includes both correlation times (*τ*_c_) and amplitude of frequency fluctuations (Δ) of the form: *c*(*t*) = ∑_*i*_Δ_*i*_^2^e^−*t*/*τ*_c*i*_^. The computed *τ*_c_ as a function of the chemical environment also shows a linear relationship with the experimental values (Fig. 4, Table 1, and the ESI†). In general, the predicted correlation times are in reasonable agreement with the experiments. In addition, NMAD/D_2_O is a particularly well studied system and has frequency maps to predict the amide I mode (*e.g.* the electric field and electrostatic potential models).^56^ Comparison of the new method with the previously reported results from frequency maps shows very similar results. Specifically, the correlation time obtained from frequency maps was ∼0.75 ps,^56^ while the new method based on GNF2-xTB predicted a value of 0.82 ps. A similar result is obtained for the NMA/DMSO system, where *τ*_c_ values of 1.6 ps and of 1.3 ps are predicted from the frequency map and the new method, respectively. In contrast, NMA in CLF presents different correlation times for the FFCF dynamics when computed using either frequency maps^28^ or the new method. In this case, the frequency map and the new method predicts *τ*_c_ values of 2.9 ps and of 1.6 ps, respectively; while the experimental result is 1.9 ps.^28^ Hence, it appears that the new method has a smaller error than the frequency map, which is likely due to the IFM method taking into account more detailed interactions affecting the frequency of the amide I mode, such as weak hydrogen bonds.^20^

**Table 1:** Central frequencies, correlation times, and fluctuation amplitudes determined from the IFM method, experimentally, and from frequency maps

Solvent	*Ṽ* (cm^−1^)		*τ*_c_ (ps)			Δ (cm^−1^)			
	IFM	Exp.	MD-IFM	Exp.	Freq. maps	IFM	Exp.	Freq. maps^28^	From IR
D_2_O	1680	1623	0.82 ± 0.03	1.2 (ref. 64)	0.76,^56^ 0.75 (ref. 56)	20.9 ± 0.3	19.75,^28^ 9 (ref. 63)	17^a^	8.2
CLF	1712	1669	1.6 ± 0.7	1.9 ± 0.1	2.9 (ref. 28)^a^	12.1 ± 0.3	13.57 (ref. 28)	8.8^a^	7.7
DMSO	1712	1668	1.3 ± 0.2	1.7 ± 0.1	1.6 (ref. 28)^a^	10.95 ± 0.08	11.55 (ref. 28)	7.8^a^	6.1
THF	1727	1684	1.02 ± 0.06	1.5 ± 0.1	N/A	9.1 ± 0.1	N/A	N/A	3.1
TOL	1747	1686	1.06 ± 0.07	1.6 ± 0.2	N/A	7.0 ± 0.2	N/A	N/A	3.4
DMSO : THF	1721	1676	1.9 ± 0.1	2.0 ± 0.2	N/A	10.2 ± 0.1	N/A	N/A	6.1
DMSO : D_2_O	1696	1646	3.8 ± 0.2	3.1 ± 0.3	N/A	14.6 ± 0.2	N/A	N/A	11.1

a Averaged value.

The goodness of the MD-IFM method for computing instantaneous frequencies was also further supported by computation of NMA amide I in other chemical environments. For example, the method predicts *τ*_c_ values of 1.02 ps and 1.06 ps for the NMA/THF and NMA/TOL systems, respectively, which are in good agreement with the experimental values of 1.5 ps (NMA/THF) and 1.6 ps (NMA/TOL). Overall, the use of the new method in single solvents allows us to demonstrate not only the validity of the MD-IFM method for predicting the dynamics of the FFCF when compared to other methods but also the transferability of the IFM method to different solvents without the need for modification or reparametrization.

The adequate predictions made by the MD-IFM method allow us to extend its application to complex molecular systems, such as mixtures of solvents. To this end, two different mixtures were selected: DMSO : THF (1 : 3 molar ratio) and DMSO : D_2_O (1 : 1 molar ratio). These two particular systems represent solvents with competitive dipole–dipole interactions as well as hydrogen bond and picosecond dynamics.^28,40,56,59,60^ The characteristic time of the FFCF for the amide I vibrational mode in these two solvents was found to be 1.9 ps (DMSO : THF) and 3.8 ps (DMSO : D_2_O), which are in very good agreement with the observed experimental values of 2.0 and 3.2 ps, respectively (see Fig. 4, Table 1, and the ESI†).

These last two cases are particularly important because of the complexity of the molecular environment or, analogously, the chemical interaction landscape. The selected molecular landscapes make it particularly challenging to parametrize a single frequency map capable of representing specific probes in various solvents. Consequently, a single frequency map is usually not capable of describing the solvatochromism of a probe on both the individual solvents and their mixtures when the solvents are very different. This is the case for DMSO and D_2_O, where not only the hydrogen bond donating and accepting capabilities are changed but also their dipoles. The correct description of both single solvents and mixtures provides strong support for the broad applicability of the new methodology in complex environments.^31,61^ Overall, it is shown that the IFM-MD methodology can be applied to compute with good agreement with the experiment not only the vibrational solvatochromism of molecules in solution (*R*^2^ = 0.874, Fig. 3) but also the FFCF decorrelation times (*R*^2^ = 0.976, Fig. 4). More importantly, this is achieved without any additional parameterization or modelling beyond the MD simulation force field.

It is derived from the data that central frequencies and correlation times of the amide I mode in different solvents are not correlated (*R*^2^ = −0.192, see the ESI†). This result demonstrates that the two vibrational observables are independent and that both are needed to obtain a correct description of the molecular system. However, the central frequencies and correlation times are not sufficient to completely determine the molecular environment observed by a molecule. A good example of the ambiguity in determining the molecular environment from vibrational observables can be seen in the cases of NMA/DMSO and NMA/CLF. In these two systems, the central frequencies for the amide I mode (1712 cm^−1^ for both) and the correlation times (1.3 ± 0.2 and 1.6 ± 0.7 ps, respectively) are similar. Therefore, these predictions, while accurate, are not sufficient to determine the chemical environment of the studied molecule and require a third observable.

Thus far, only the central frequencies and correlation times have been used to assess the molecular environment of a molecule using IR spectroscopy. Another important parameter that defines the line shape of a vibrational transition is the amplitude of the frequency fluctuations. This vibrational observable contains information about the ensemble of molecular environments that the molecule can observe thermally. Consequently, the amplitudes of frequency fluctuations (Δ) are strongly dependent on the chemical nature of the system.^27^ Experimentally, these amplitudes are obtained by modelling the FTIR and 2DIR spectra.^62^ Particularly, the computed amplitudes are found to be 12.1 ± 0.3, 10.95 ± 0.08 and 20.9 ± 0.3 cm^−1^ for NMA in CLF, DMSO and D_2_O, respectively, and compare very well with those derived from the experiment: 13.57 cm^−1^, 11.55 cm^−1^, and 19.75 cm^−1^, respectively.^28^

Nonetheless, it is important to note that the NMA in D_2_O experimental Δ has been reported by others to be 9 cm^−1^.^63^ Therefore, it was excluded from the overall analysis of the frequency fluctuation amplitude.

**Fig. 5:**
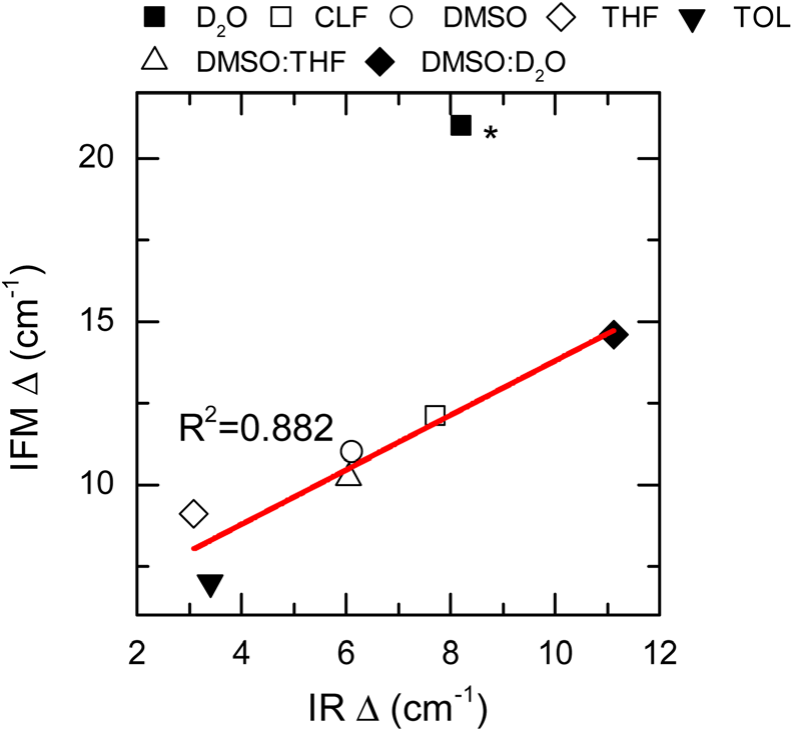
Frequency fluctuation amplitudes (Δ) calculated by the IFM *vs.* determined from the IR spectra (data points). Linear fit corresponds to the red line. Star (*) NMA in the D_2_O Δ value was not included into the fit.

The IFM-based amplitudes of the remaining systems were compared with the experimental values by using a more approximate method to compute the fluctuation amplitudes from the FTIR and 2DIR spectra.^62^ The experimental amplitudes, Δ, show a good agreement with those obtained by the IFM method (Fig. 5 and Table 1). The correlation between the experiment and computation is very good, as seen by the linear correlation between the experimental and computed values (*R*^2^ = 0.882). Interestingly, the amplitude for the NMA/TOL system has the largest deviation from the linear trend observed for the other samples. In the case of NMA/TOL, the result is not surprising, given that the method is not particularly good at predicting the vibrational solvatochromism of the same system, as previously shown. Nonetheless, the high Pearson correlation coefficient between the experimental and computation amplitudes indicates that the IFM-based method predicts reasonably well the amplitudes of the frequency fluctuations in different systems. More importantly, the strong correlation between the experiment and theory shows that the IFM method captures more detailed interactions, which directly translates into more accurate frequency fluctuations, even when compared to other maps.

Overall, it was demonstrated that the method based on the IFM frequencies produces a reliable prediction of the solvatochromism (*ω*_0_), dynamics (*τ*_c_) and distribution of environments (Δ) from different samples without the need for reparametrization, which is usually required when using frequency maps. These metrics, obtained for a molecule (NMA) solvated in pure solvents and their mixtures, describe the effect of the dynamics and interactions of the molecular environment on the investigated molecule. Therefore, the method presented here allows unambiguous assignment of experimental observables in the vibrational spectra (linear and non-linear), unlocking the full potential of vibrational observables to create molecular maps capable of describing the interaction landscape of complex molecular systems.

## Conclusions

A new method for frequency calculation is presented. This method takes advantage of the existing and new computational tools (MD simulations and semi-empirical methods), as well as of current computational power to obtain the frequencies of solvated molecules in large clusters. The new methodology in conjunction with MD simulations allows us to determine vibrational solvatochromism, dynamics, and distribution of environments. The new method is successfully tested on a well-studied system: *N*-methylacetamide in polar solvents and their mixtures. In addition, the transferability of the method to more complex binary solutions was examined. The correct description of the vibrational solvatochromism in both simple and complex chemical environments shows the utility of the method to predict the location of a probe in a solution. Moreover, it is observed that the combination of frequencies, decorrelation times and fluctuation amplitudes is sufficient to fully characterize a specific system. The use of such a tool is particularly important for vibrational spectroscopy to unlock the full potential of the IR methodology when probing highly heterogeneous environments.

## Author contributions

L. X. S.-M.: methodology, data curation, validation, formal analysis, investigation, writing – original draft, visualization; J. F. G.: methodology, writing – original draft, writing – review and editing, supervision; D. G. K.: conceptualization, methodology, writing – review and editing, resources, supervision, project administration, funding acquisition.

## Conflicts of interest

There are no conflicts to declare.

## Supplementary Material

SC-015-D4SC03219E-s001

## Data Availability

The data that support the findings of this study are available from the corresponding author upon reasonable request.
