# Correction: A new computational methodology for the characterization of complex molecular environments using IR spectroscopy: bridging the gap between experiments and computations

**DOI:** 10.1039/d4sc90187h

**Published:** 2024-11-05

**Authors:** Laura X. Sepulveda-Montaño, Johan F. Galindo, Daniel G. Kuroda

**Affiliations:** a Department of Chemistry, Louisiana State University Baton Rouge Louisiana 70803 USA dkuroda@lsu.edu; b Department of Chemistry, Universidad Nacional de Colombia Sede Bogotá Bogotá 111321 Colombia

## Abstract

Correction for ‘A new computational methodology for the characterization of complex molecular environments using IR spectroscopy: bridging the gap between experiments and computations’ by Laura X. Sepulveda-Montaño *et al.*, *Chem. Sci.*, 2024, **15**, 14440–14448, https://doi.org/10.1039/D4SC03219E.

The grant number given in the original version of this manuscript was incorrect. The corrected grant number is CHE-1751735.

The full acknowledgements should be as follows:

L. X. S.-M. and D. G. K. acknowledge the financial support from the National Science Foundation (CHE-1751735) and the computer time from the High Performance Computing Center at Louisiana State University and the Louisiana Optical Network Initiative (LONI). D. G. K. would like to thank LSS, his work has been a tremendous inspiration to him.

The Royal Society of Chemistry apologises for these errors and any consequent inconvenience to authors and readers.

