# Supplementary material for: A new computational methodology for the characterization of complex molecular environments using IR spectroscopy: bridging the gap between experiments and computations
Source: Chem Sci. 2024 Aug 13;15(35):14440–8. doi: 10.1039/d4sc03219e (PMC11328912; doi:10.1039/d4sc03219e)
Supplement: SC-015-D4SC03219E-s001 [file SC-015-D4SC03219E-s001.pdf]

Supplementary Information (SI) for Chemical Science.
This journal is © The Royal Society of Chemistry 2024

Supplementary Information

A New Computational Methodology for the Characterization of Complex Molecular Environments Using IR Spectroscopy: Bridging the Gap between Experiments and Computations

Laura X. Sepulveda-Montaño, [a] Johan F. Galindo, [b] and Daniel G. Kuroda [*, a]

[a] Department of Chemistry, Louisiana State University, Baton Rouge, Louisiana 70803, United States.

Emails: lsepcl1@lsu.edu (L. X. S-M), and dkuroda@lsu.edu (D.G.K.)

[b] Department of Chemistry, Universidad Nacional de Colombia sede Bogotá, 111321 Bogotá, Colombia.

Email: jfgalindoc@unal.edu.co(J.F.G.)

Experimental procedures

Sample preparation

NMA (TCI AMERICA, 99.0%), DMSO (Sigma Aldrich, 99.9%), THF (Fisher, 99.9%), CLF (Sigma Aldrich 99+%), TOL (Sigma Aldrich 99.8%), and D_2O (Sigma Aldrich 99.9%) were used as received. All solutions had a concentration of ~100mM, except NMA in TOL and DMSO:THF (1:3) which were prepared at ~10Mm. All samples, with the exception of NMA in D_2O and in DMSO:D_2O (1:1), were prepared and stored in a nitrogen-flushed glovebox.

Linear IR spectroscopy

Linear IR frequency measurements were taken with a Bruker Tensor 27 equipped with a liquid nitrogen-cooled narrow-band MCT detector. The spectrum was averaged over 40 scans with a resolution of 0.5 cm^{-1} for samples enclosed in CaF_2 windows with 100μm path length.

2DIR Spectroscopy

The 2DIR experimental setup has been formerly reported.[1, 2] Therefore, only a brief description is presented here. A Spectra-Physics Spitfire ACE Ti:sapphire amplifier with a repetition rate of 5 kHz paired with an OPA-800C generates broadband infrared pulses. Each of them is split into four equal beams, three of which (with wave vectors k_1, k_2, and k_3) are directed to the sample at different times in a box card configuration. [3] The time intervals between the pulses are set via computer-controlled translational stages and are denominated τ (time between pulse 1 and 2), T_w (time between pulse 2 and 3), and t (time between pulse 3 and photon echo). The photon echo (PE) is produced in the $-k_1 + k_2 + k_3$ direction and heterodyned with the fourth initial pulse or Local Oscillator (LO). The signal is detected with a Nitrogen-cooled 64 element MCT detector as a function of (τ, T_w, t) a double Fourier transform is used to obtain the 2DIR spectrums as a function of (ω_τ, T_w, ω_t). [4] Here, data was collected for T_w from 0 to 4 ps with 250 fs time steps, with the LO preceding the PE by ~0.8 ps and τ was scanned from -3.5 to 3.5 ps every ~5fs.

Computational Cost

The computational cost between a higher theory level, DFT (B3LYP/6-311++g[d,p]), and the GFN2-xTB semiempirical method when using the IFM approach was evaluated using ten clusters, each containing ten water molecules and one NMA. These calculations were carried out on a node computer with 64 processors.

Table S1. Wall-clock time of the IFM calculation for two different levels of theory GFN2-xTB and DFT.

Frame	GFN2-xTB time (s)	DFT time (s)	Frame	GFN2-xTB time (s)	DFT time (s)
1	1.2	6440.5	6	1.4	6205.4
2	1.2	8044.4	7	1.1	6719.1
3	1.1	3657.2	8	1.7	9095.3
4	0.7	7079.7	9	1.2	3414.8
5	1.1	6424.4	10	1.1	4347.6
			<t>	1.2	6142.8

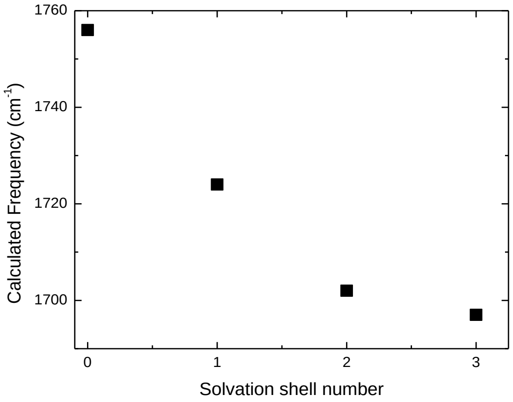

Figure S1. NMA in D_2OAmide I mode in function of the number of solvation shells.

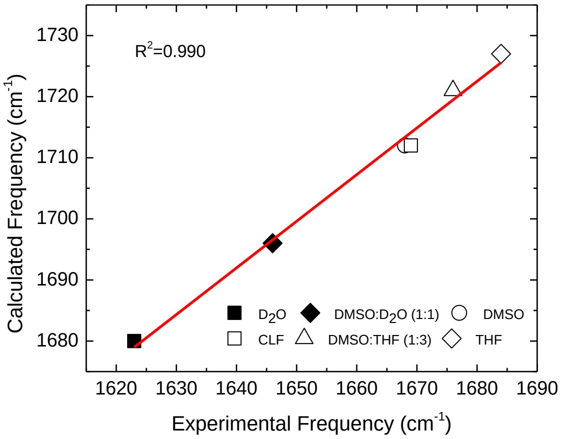

Figure S2.Solvatochromic shift for the NMA amide I mode determined through the GFN2-xTB method vs determined experimentally by FTIR, for all solvents treated in the main text except for TOL.

Frequency distributions

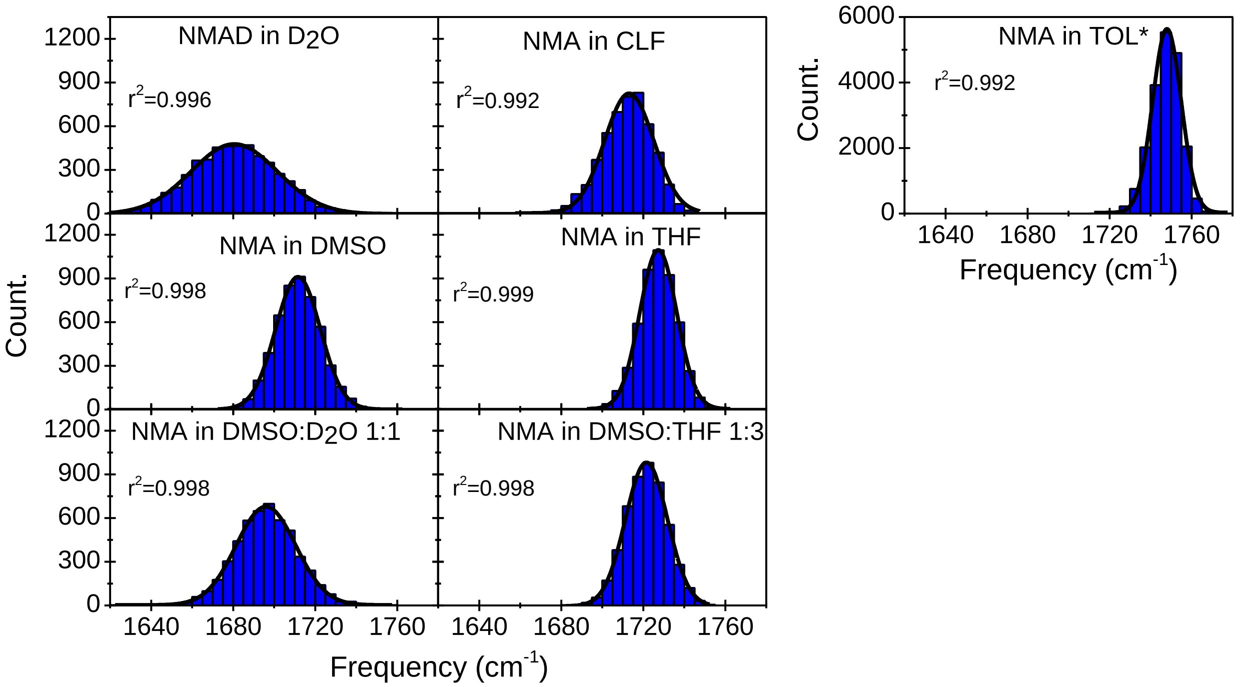

Figure S3. Frequency distributions for the amide I mode of NMA in all chemical environments considered. *The NMA in TOL system was run for 1ns to improve averaging of the FFCF-OD calculation.

Table S2. Fit parameters for the amide I mode in all chemical environments considered for the frequency distributions of Figure S2.

**Solvent**	**Average of distribution (cm^{-1})**	**Δ(cm^{-1})**	**Amplitude**
**D_2O**	1680.5±0.3	20.9±0.3	476±5
**CLF**	1713.1±0.3	12.1±0.3	824±16
**DMSO**	1711.6±0.1	10.95±0.08	910±6
**THF**	1727.0±0.1	9.1±0.1	1093±9
**DMSO:D_2O (1:1)**	1696.0±0.2	14.6±0.2	677±6
**DMSO:THF (1:3)**	1722±0.1	10.2±0.1	983±7
**TOL***	1748.0±0.2	7.0±0.2	5623±141

*The NMA in TOL system was run for 1ns to improve averaging of the FFCF-OD calculation.

Correlation functions

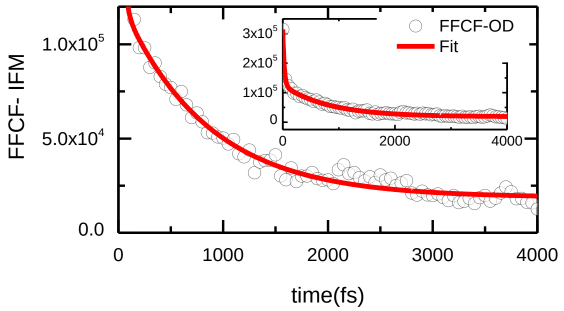

Figure S4. FFCF-OD of NMAD in D2O, and biexponential decay fit (red line). Inset presents the complete amplitude of the FFCF-OD.

Table S2. NMA in D_2O, fit parameters for computational and experimental FFCF, the FFCF graph can be found in the main manuscript.

**Method**	A_1	τ_1**(ps)**	A_2	τ_2**(fs)**	Y_0	R^2
**FFCF-OD**	$1.1\pm0.02(_x10^5)$	0.82 ± 0.03	$1.9\pm0.05(_x10^5)$	26 ± 2	$1.9\pm0.09(_x10^4)$	0.992
**NLS.[5]**	0.39	1.2	-	-	-	-

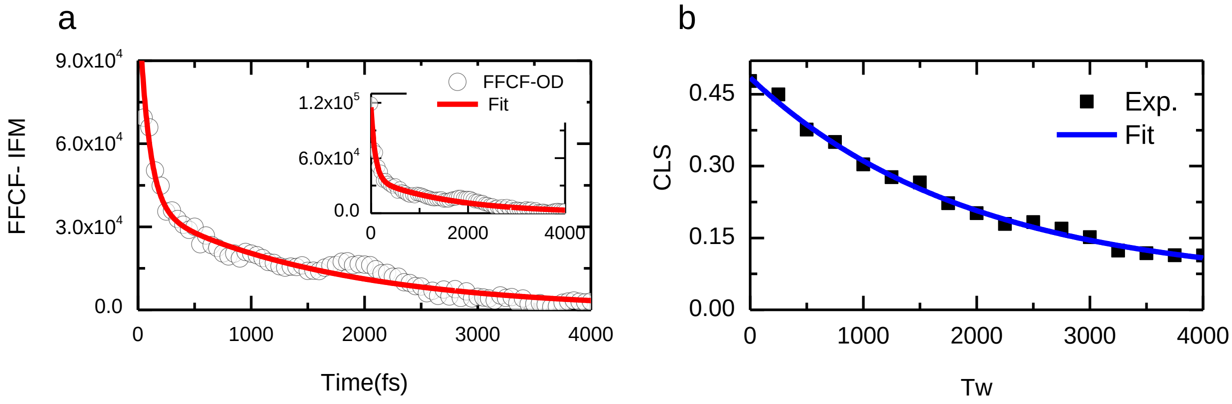

Figure S5. Left panel (a): FFCF-OD of NMA in CLF, and biexponential decay fit (red line). Inset presents the complete amplitude of the FFCF-OD. Right panel (b): Time decay of the FFCF determined from 2DIR using the CLS method where the black squares represent the CLS at every waiting time and a fit with an exponential decay (blue line).

Table S3. NMA in CLF fit parameters for the FFCF-OD method and CLS fit.

**Method**	A_1	τ_1**(ps)**	A_2	τ_2**(fs)**	Y_0	R^2
**FFCF-OD**	$3.7\pm0.2(_x10^4)$	1.6 ± 0.7	$7.9\pm0.3(_x10^4)$	89 ± 7	0	0.976
**CLS**	0.43 ± 0.01	1.9 ± 0.1	-	-	0.05 ± 0.02	0.993

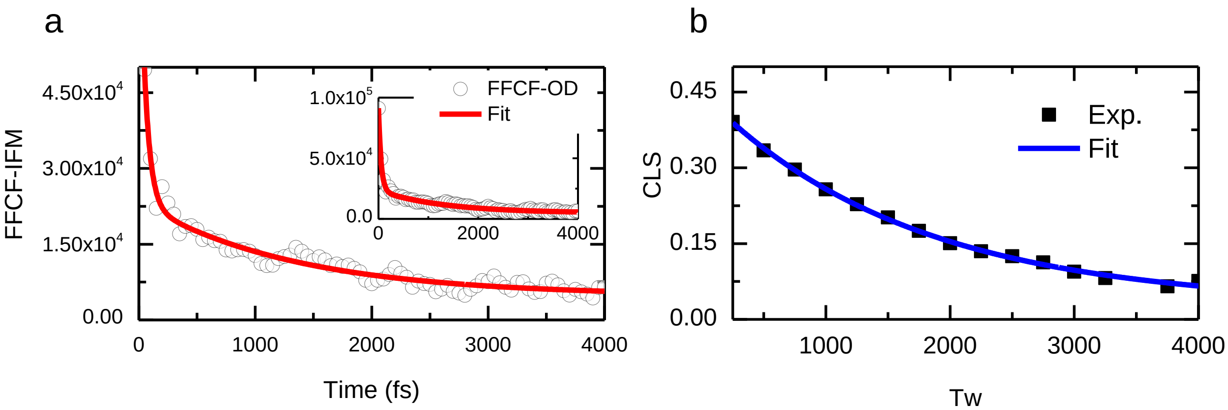

Figure S6. Left panel (a): FFCF-OD of NMA in DMSO, and biexponential decay fit (red line) inset present the complete amplitude of the FFCF-OD. . Right panel (b): Time decay of the FFCF determined from 2DIR using the CLS method where the black squares represent the CLS at every waiting time. Tw=3500fs was discarded due to scatter.

Table S4.NMA in DMSO fit parameters for the FFCF-OD method and CLS fit.

**Method**	**A_1**	**τ_1(ps)**	**A_2**	**τ_2(fs)**	**Y_0**	**R^2**
**FFCF-OD**	1.86±0.07($_x10^4$)	1.3±0.2	6.8±0.2($_x10^5$)	53±0.3	4.8±0.6($_x10^3$)	0.985
**CLS**	0.42±0.01	1.7±0.1	-	-	0.03±0.01	0.997

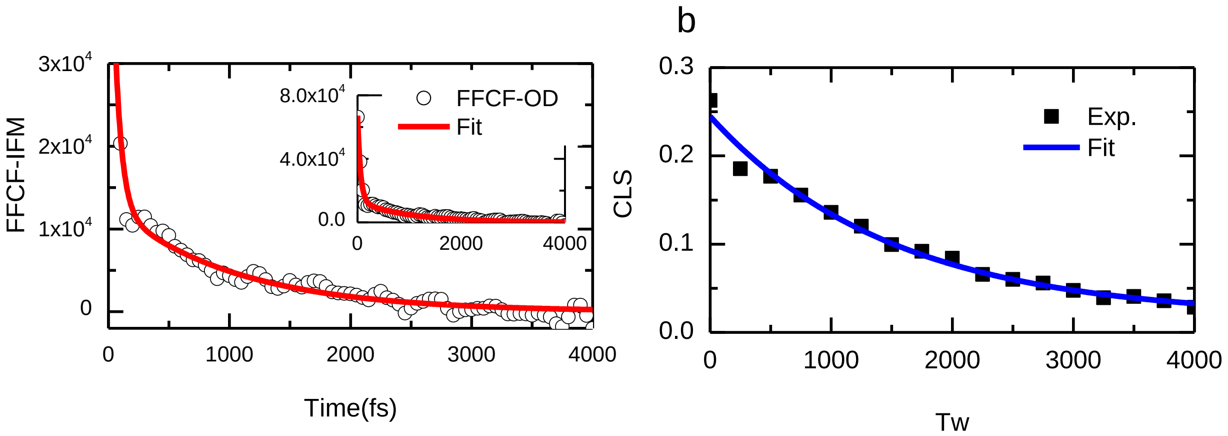

Figure S7. Left panel (a):FFCF-OD of NMA in THF, and biexponential decay fit (red line) inset present the complete amplitude of the FFCF-OD. Right panel (b): Time decay of the FFCF determined from 2DIR using the CLS method where the black squares represent the CLS at every waiting time.

Table S5. NMA in THF fit parameters for the FFCF-OD method and CLS fit.

**Method**	**A_1**	**τ_1 (ps)**	**A_2**	**τ_2 (fs)**	**Y_0**	**R^2**
**FFCF-OD**	1.3±0.01($_x10^4$)	1.02±0.06	5.4±0.1($_x10^4$)	57±3	0	0.987
**CLS**	0.23±0.01	1.5±0.1	-	-	0.02±0.01	0.982

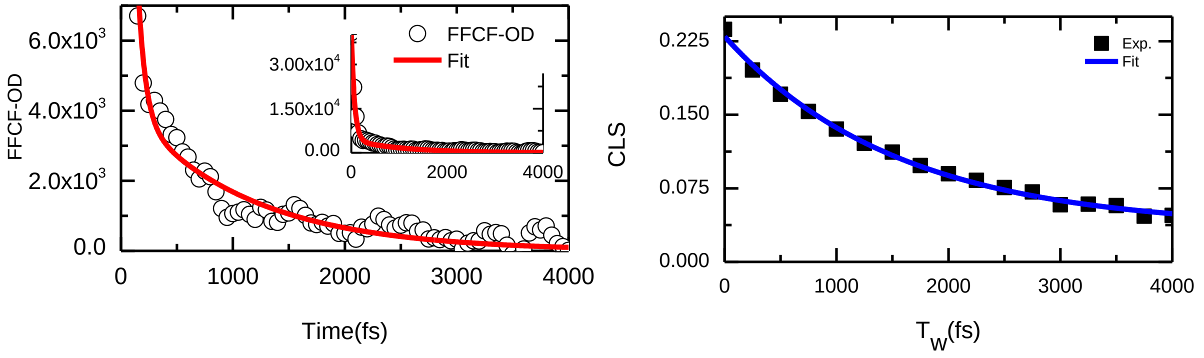

Figure S8. Left panel (a): FFCF-OD of NMA in TOL, and biexponential decay fit (red line) inset present the complete amplitude of the FFCF-OD. Right panel (b): Time decay of the FFCF determined from 2DIR experiments using the CLS method where the black squares represent the CLS at every waiting time.

Table S6. NMA in TOL fit parameters for the FFCF-OD method and CLS fit.

**Method**	**A_1**	**τ_1(ps)**	**A_2**	**τ_2(fs)**	**Y_0**	**R^2**
**FFCF-OD**	4.3±0.3(x10^3)	1.06±0.07	3.67±0.04(x10^4)	67±2	0	0.995
**CLS**	0.0195±0.004	1.6±0.1	-	-	0.034±0.004	0.995

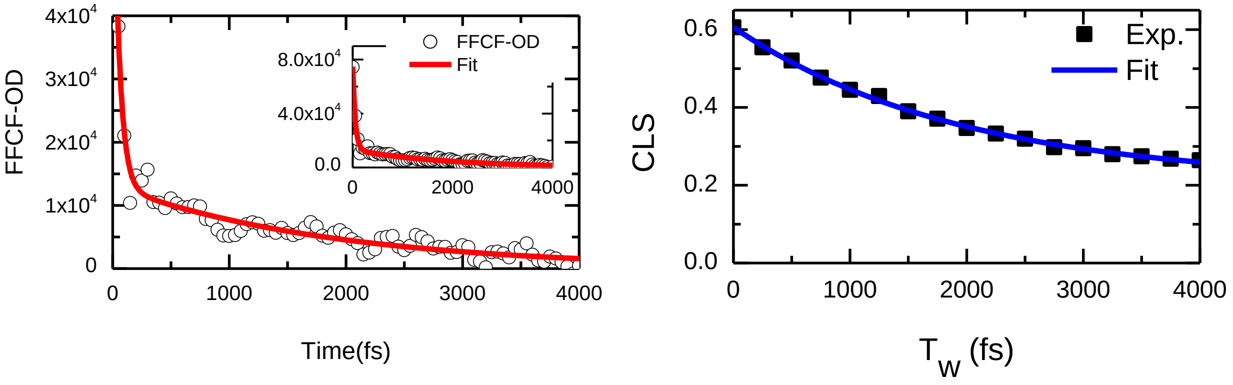

Figure S9. Left panel (a): FFCF-OD of NMA in DMSO:THF (1:3), and biexponential decay fit (red line) inset present the complete amplitude of the FFCF-OD. Right panel (b): Time decay of the FFCF determined from 2DIR experiments using the CLS method where the black squares represent the CLS at every waiting time.

Table S7. NMA in DMSO:THF 1:3 fit parameters for the FFCF-OD method and CLS fit.

**Method**	**A_1**	**τ_1(ps)**	**A_2**	**τ_2(fs)**	**Y_0**	**R^2**
**FFCF-OD**	1.3±0.1(x10^4)	1.9±0.1	6.2±0.2(x10^4)	53±3	0	0.978
**CLS**	0.40±0.01	2.0±0.1	-	-	0.21±0.02	0.998

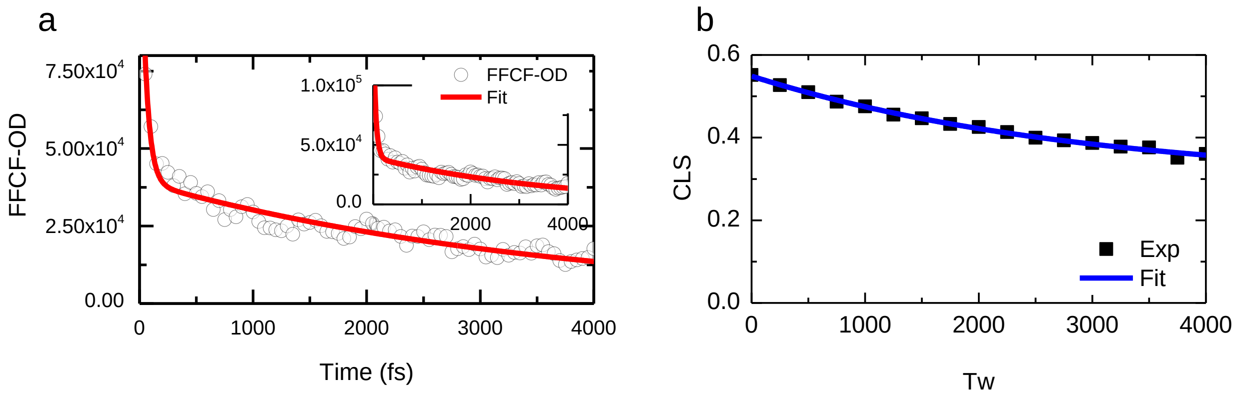

Figure S10. Left panel (a): FFCF-OD of NMA in DMSO:D_2O (1:1), and biexponential decay fit (red line) inset present the complete amplitude of the FFCF-OD. Right panel (b): Time decay of the FFCF determined from 2DIR experiments using the CLS method where the black squares represent the CLS at every waiting time which was fitted to an exponential decay (blue line).

Table S8. NMAD in DMSO:D_2O (1:1) fit parameters for the FFCF-OD method and CLS fit.

Method	A_1	τ_1(ps)	A_2	τ_2(fs)	Y_0	R^2
**FFCF-OD**	3.94±0.9(x10^4)	3.8±0.2	1.11±0.3(x10^5)	49±3	0	0.977
**Exp**	0.26±0.01	3.0±0.3	-	-	0.29±0.01	0.995

Correlation between observables

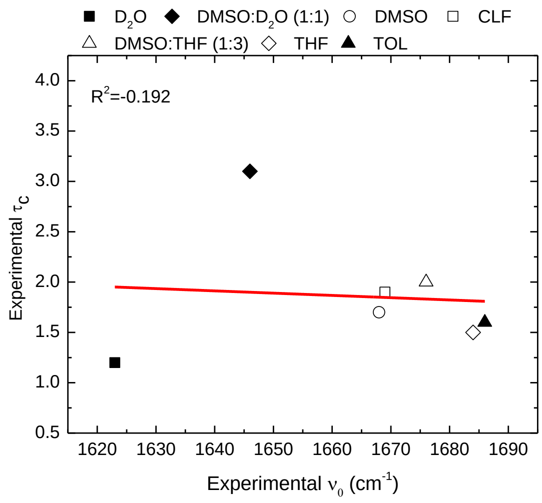

Figure S11. Relation between experimental frequencies and correlation times for the NMA in different chemical environments.

References

1. Asplund, M., M.T. Zanni, and R.M. Hochstrasser, *Two-dimensional infrared spectroscopy of peptides by phase-controlled femtosecond vibrational photon echoes.* Proceedings of the National Academy of Sciences, 2000. **97**(15): p. 8219-8224.
2. Kim, Y.S. and R.M. Hochstrasser, *Applications of 2D IR spectroscopy to peptides, proteins, and hydrogen-bond dynamics.* The Journal of Physical Chemistry B, 2009. **113**(24): p. 8231-8251.
3. Hamm, P. and M. Zanni, *Concepts and methods of 2D infrared spectroscopy*. 2011: Cambridge University Press.
4. Kim, Y.S., J. Wang, and R.M. Hochstrasser, *Two-dimensional infrared spectroscopy of the alanine dipeptide in aqueous solution.* The journal of physical chemistry B, 2005. **109**(15): p. 7511-7521.
5. Ghosh, A. and R.M. Hochstrasser, *A peptide's perspective of water dynamics.* Chemical physics, 2011. **390**(1): p. 1-13.
